# Arthroscopic Resection of a Tenosynovial Giant Cell Tumor in the Wrist: A Case Report

**DOI:** 10.1097/MD.0000000000001887

**Published:** 2015-10-23

**Authors:** Young-Keun Lee, Youngshin Han, Malrey Lee

**Affiliations:** From the Department of Orthopedic Surgery, Chonbuk National University Hospital, Jeonju (Y-KL); Department of Computer Engineering, Sungkyul University, Anyang (YH); and Research Center for Advanced Image and Information Technology, School of Electronics and Information Engineering, Chonbuk National University, Jeonju, South Korea (ML).

## Abstract

The treatment for giant cell tumors of the tendon sheath is surgical therapy, but surgical recurrence rates were reported to be as high as 50% in some cases. Therefore, complete radical excision of the lesion is the treatment of choice. If the tumor originates from the joint, it is important to perform capsulotomy. Here, the authors report the first case of successful treatment of a localized intra-articular giant cell tumor in the wrist by arthroscopic resection.

A 28-year-old right-handed woman visited the clinic because of left wrist ulnar-side pain, which had been aggravated during the previous 15 days. Vague ulnar-side wrist pain had begun 2 years ago. When the authors examined the patient, the wrist showed mild swelling on the volo-ulnar aspect and the distal radioulnar joint, as well as volar joint line tenderness. She showed a positive result on the ulnocarpal stress test and displayed limited range of motion. Magnetic resonance imaging revealed an intra-articular mass with synovitis in the ulnocarpal joint. Wrist arthroscopy was performed using standard portals under regional anesthesia. The arthroscopic findings revealed a large, well-encapsulated, yellow lobulated soft-tissue mass that was attached to the volar side of the ulnocarpal ligament and connected to the extra-articular side. The mass was completely excised piece by piece with a grasping forceps. Histopathologic examination revealed that the lesion was an intra-articular localized form of a tenosynovial giant cell tumor.

At 24-month follow-up, the patient was completely asymptomatic and had full range of motion in her left wrist, and no recurrence was found in magnetic resonance imaging follow-up evaluations.

The authors suggest that the arthroscopic excision of intra-articular giant cell tumors, as in this case, may be an alternative method to open excisions, with many advantages.

## INTRODUCTION

Giant cell tumor of the tendon sheath is the second most common tumor found in the hand. It occurs almost exclusively in the hand, though it has also been reported in other parts of the body.^1–4^ Moore et al^2^ used the term localized nodular tenosynovitis to provide a more accurate description of the clinical appearance of the tumor when it is found in the hand. Giant cell tumors of the tendon sheath have a favorable prognosis because they are not considered to be malignant lesions.^5^ These tumors have less frequent association with joints. When association with a joint occurs, the tumor usually grows outside and extends into or near the joint. Here, we report the first case of successful treatment of a localized intra-articular giant cell tumor in the wrist by arthroscopic resection.

## CONSENT

The patient signed informed consent for the publication of this case report and any accompanying images. Ethical approval of this study was waived by the ethics committee of Chonbuk National University Hospital because it was a case report and there were fewer than 3 patients.

### Case Report

A 28-year-old right-handed woman visited the clinic because of left wrist ulnar-side pain, which had been aggravated during the previous 15 days. Vague ulnar-side wrist pain had begun 2 years ago, and she reported that she was an office worker and had worked with a computer 5 days a week for 8 hours a day for the last 7 years. Her height was 163 cm and weight was 48 kg. She was diagnosed as having tenosynovitis at another clinic and was treated with physical therapy, medication, and steroid injection, but her symptoms did not improve. The patient had no trauma history or evidence of systemic diseases.

When we examined the patient, the wrist showed mild swelling on the volo-ulnar aspect and the distal radioulnar joint, as well as volar joint line tenderness. She showed a positive result on the ulnocarpal stress test and displayed limited range of motion. The flexion and extension angles of the left wrist were 60° and 30°, respectively (with 70° and 60° in the right). The radial and ulnar deviation angles in the left wrist were 20° and 30°, respectively (with 20° and 45° in the right).

**FIGURE 1:**
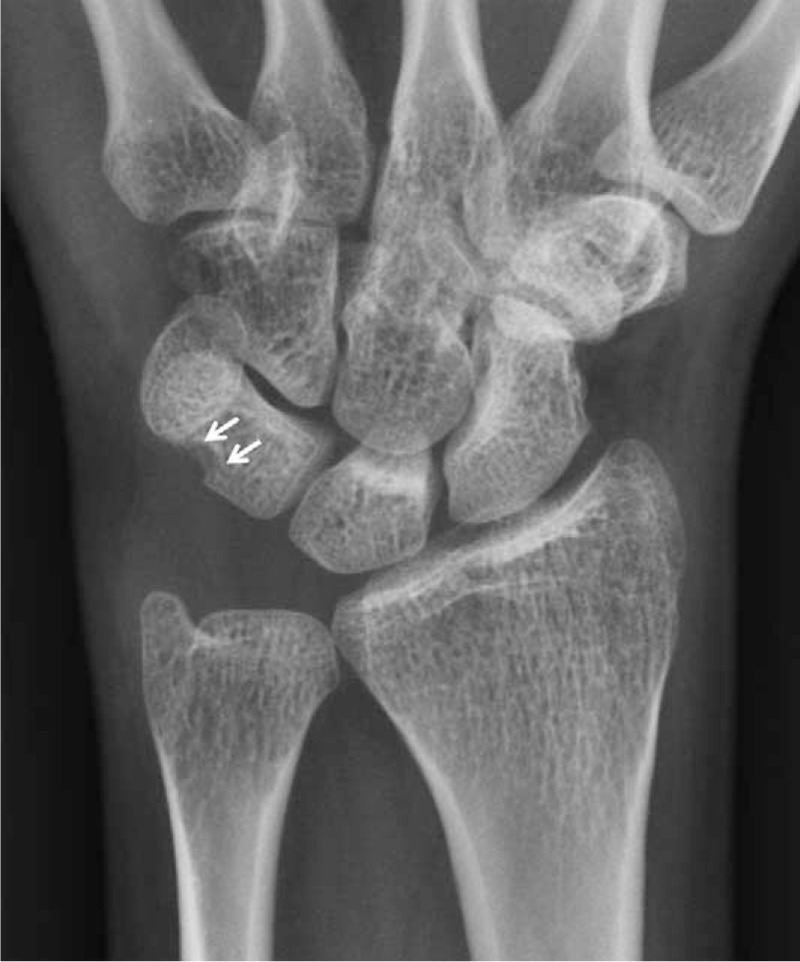
Anteroposterior radiograph of the left wrist, showing pressure bone erosion with sclerotic margin in the triquetrum (arrows).

**FIGURE 2:**
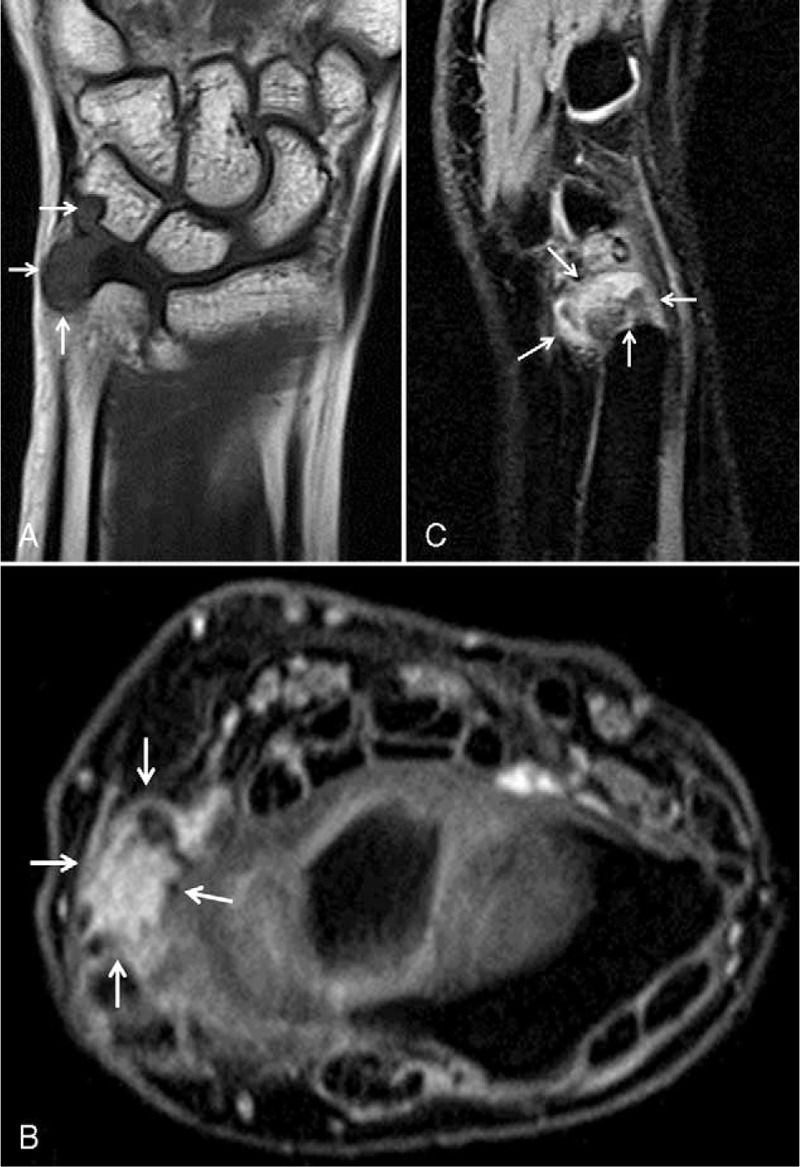
A, Coronal T1-weighted magnetic resonance image of the left wrist, showing a well-defined mass that is slightly hypointense relative to the skeletal muscle and is compressing the triquetrum (arrows). B, Axial fat-suppressed T1-weighted postcontrast. C, Sagittal fat-suppressed T1-weighted postcontrast magnetic resonance images of the left wrist, showing a marked heterogeneous enhancement of the mass (arrows).

**FIGURE 3:**
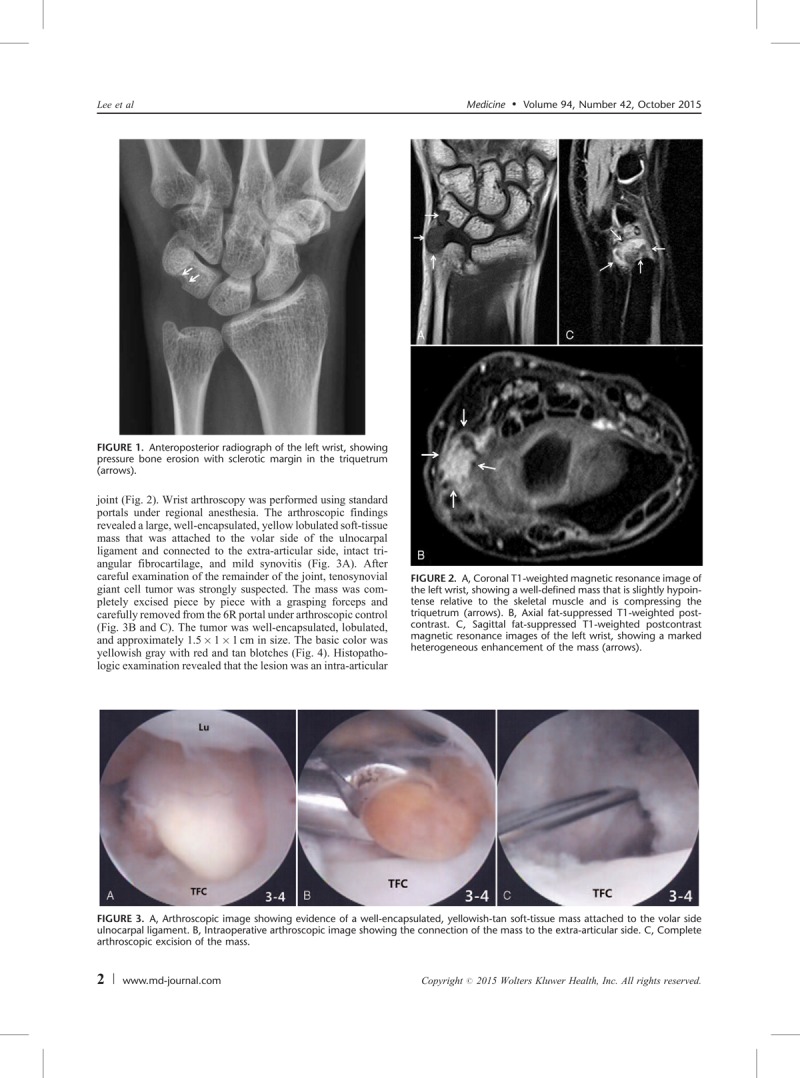
A, Arthroscopic image showing evidence of a well-encapsulated, yellowish-tan soft-tissue mass attached to the volar side ulnocarpal ligament. B, Intraoperative arthroscopic image showing the connection of the mass to the extra-articular side. C, Complete arthroscopic excision of the mass.

**FIGURE 4:**
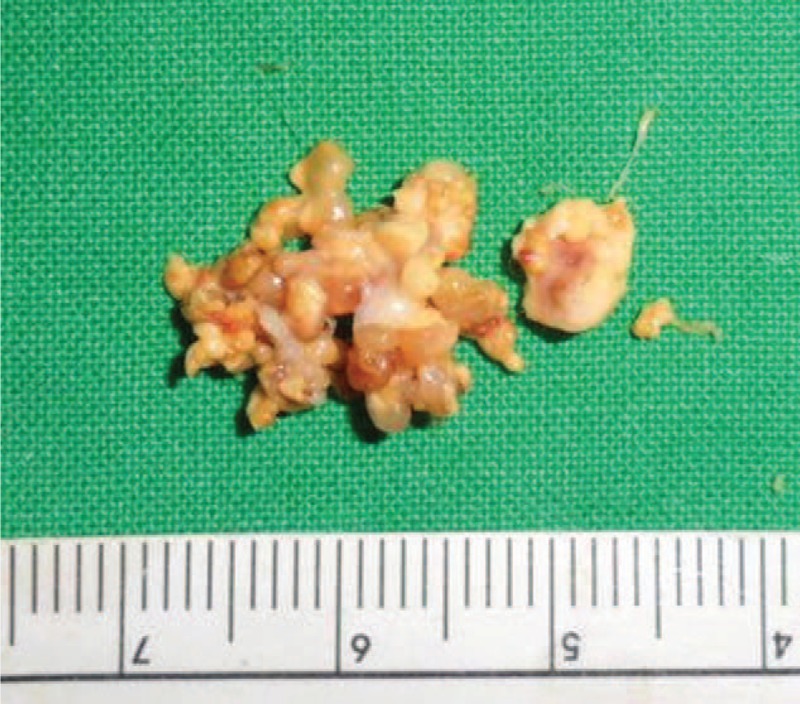
The size of the tumor was 1.5 × 1 × 1 cm. The aspect was yellowish in color and mottled with tan blotches.

**FIGURE 5:**
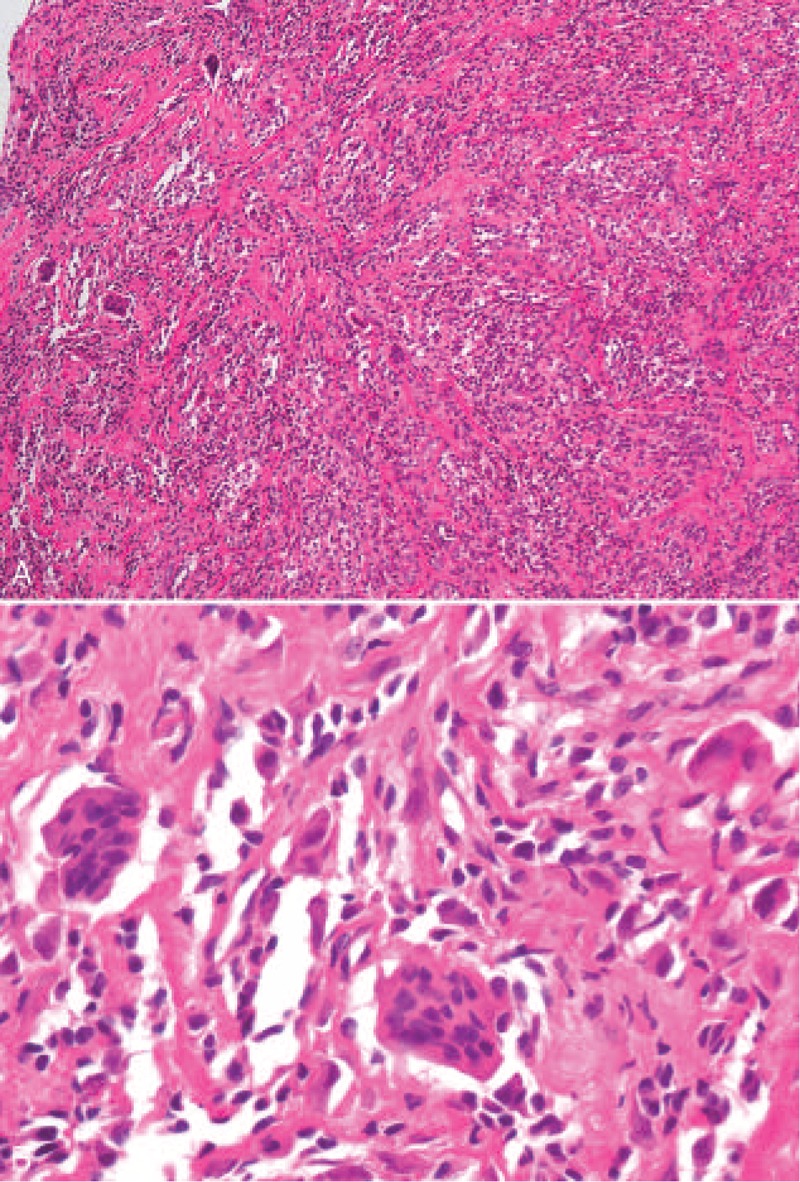
A-B, Microscopic appearances of the tumor, showing a polymorphic infiltrate of histocytes and a multinucleated giant cell embedded in the fibrotic stroma. Xanthomatous foamy cells and lymphocytes were also noted, but there was no mitotic figure and no villous projection of the synovium (hematoxylin and eosin, original magnification ×100, ×400).

Initial plain wrist radiograph showed pressure bone erosion with sclerotic margin in the triquetrum and mild soft-tissue swelling on the ulnar side (Fig. 1). Magnetic resonance imaging revealed an intra-articular mass with synovitis in the ulnocarpal joint (Fig. 2). Wrist arthroscopy was performed using standard portals under regional anesthesia. The arthroscopic findings revealed a large, well-encapsulated, yellow lobulated soft-tissue mass that was attached to the volar side of the ulnocarpal ligament and connected to the extra-articular side, intact triangular fibrocartilage, and mild synovitis (Fig. 3A). After careful examination of the remainder of the joint, tenosynovial giant cell tumor was strongly suspected. The mass was completely excised piece by piece with a grasping forceps and carefully removed from the 6R portal under arthroscopic control (Fig. 3B and C). The tumor was well-encapsulated, lobulated, and approximately 1.5 × 1 × 1 cm in size. The basic color was yellowish gray with red and tan blotches (Fig. 4). Histopathologic examination revealed that the lesion was an intra-articular localized form of a tenosynovial giant cell tumor. There was no mitotic figure or villous projection of the synovium (Fig. 5).

Postoperatively, the patient was placed in a short arm splint. Two weeks after surgery, the patient was seen back at the clinic for a wound check, suture removal, and further immobilization in a removable short arm brace for an additional 4 weeks. After that, she was seen back at the clinic every 2 weeks to check the condition of the left wrist. At 6 weeks after the surgery, gradually increasing the active range of motion of left wrist was recommended. At 8 weeks after the surgery, there was no pain or swelling in her left wrist, and the flexion and extension angles were 60° and 30°, respectively. After that, she was seen back at the clinic every 6 months to check the condition of the left wrist. At the 14-month follow-up, the wrist showed no swelling on the volo-ulnar aspect and no joint line tenderness. The flexion and extension angles were 75° and 75°, respectively, and the radial and ulnar deviation angles were 35° and 35°, respectively. The wrist grip strength was 16 kg (20 kg in the right). The visual analogue scale for pain was 0 at rest (preoperation, it was 8).

**FIGURE 6:**
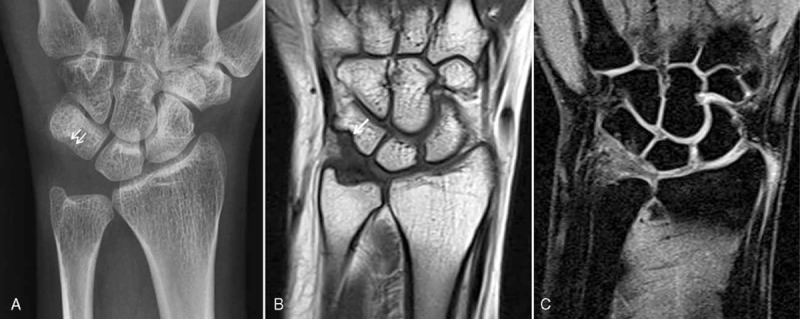
A, Anteroposterior radiograph shows no more progress of bone erosion in the triquetrum (arrows) in the 24-month follow-up magnetic resonance image of the left wrist after excision. B, Coronal T1-weighted image shows no evidence of nodular lesion to suspect a recurred mass. Note the erosion of the triquetrum from the compression of the previous mass (arrow). C, Coronal fat-suppressed T2-weighted image reveals postoperative fibrotic change in the triangular fibrocartilage complex. There is no intra-articular of the left wrist at 24-month follow-up, showing the complete lack of recurrence of the giant cell tumor.

At 24-month follow-up, the patient was completely asymptomatic and had full range of motion in her left wrist, and no recurrence was found on plain radiograph or magnetic resonance image follow-up evaluations (Fig. 6).

## DISCUSSION

The treatment for giant cell tumors of the tendon sheath is surgical therapy, but surgical recurrence rates were reported to be as high as 50% in some cases, and as low as 5% after primary excision.^1–4,6^ Even higher occurrence rates are typically observed upon subsequent re-excision.^6^ Therefore, complete radical excision of the lesion is the treatment of choice. If the tumor originates from the joint, it is important to perform capsulotomy to inspect the joint and debride any pigmented tissue within the joint. The requirement for a large incision carries the risk of infection, necrosis, or a mechanically alterable scar. In some patients, this type of complete excision may also involve the sacrifice of certain tissues, such as tendon or portions of the bone in order for the lesion to be sufficiently eradicated. Therefore, in cases of intra-articular type tumors, removal can be accomplished noninvasively through arthroscopy without resection of the affected joint. This can reduce complications and allow for rehabilitation to be started early.

Many articles have reported the successful treatment of tenosynovial giant cell tumors in the knee, ankle, and hip with arthroscopy.^7–12^ These reports have described the advantages of using this procedure.^7,9,10^ In addition, a comprehensive assessment of the joint and associated disorders can be made with simultaneous treatment during arthroscopy. The benefits include small, nonproblematic scars, reduced postoperative pain, and earlier rehabilitation. Although arthroscopic wrist surgery emerged from the stage of ablative surgery to become reparative surgery, it is moving toward the territory of reconstructive surgery.^13^ To the best of our knowledge, there have been no other reports of the arthroscopic resection of giant cell tumor in the wrist to date.

In the case of our patient, a portion of the tumor was located at the ulnocarpal joint, and the rest of the tumor was located in the extra-articular area, so it was very difficult to completely remove with the arthroscopic technique alone. The giant cell tumor was connected, so the intra-articular portion of the tumor, however, was removed by grasping forceps, and the extra-articular portion of the tumor was removed by pulling it into the joint. During the operation, the assistant pushed the extra-articular portion of the tumor into the joint with finger pressure, so it could be removed via arthroscopy.

## CONCLUSIONS

We suggest that the arthroscopic excision of intra-articular giant cell tumors, as in this case, may be an alternative method to open excision, with many advantages.
